# Modulation of Mast Cell Activation via MRGPRX2 by Natural Oat Extract

**DOI:** 10.3390/ijms27010334

**Published:** 2025-12-28

**Authors:** Susanne Kaesler, Désirée Argiriu, Shyami M. Kandage, Karla Schönfeldt, Shalva Lekiashvili, Ceren N. Dengiz, Neslim Ercan, Caterina Iuliano, Martina Herrmann, Maria Reichenbach, Dominik Cichowski, Magda Babina, Miriam Hils, Martin Köberle, Tilo Biedermann

**Affiliations:** 1 Department of Dermatology and Allergology, School of Medicine and Health, Technical University of Munich, 80802 Munich, Germany; susanne.kaesler@tum.de (S.K.); desiree.argiriu@tum.de (D.A.); shyami.kandage@tum.de (S.M.K.); karla.schoenfeldt@gmx.de (K.S.); shalva.lekiashvili@tum.de (S.L.); cerennazdengiz@gmail.com (C.N.D.); neslim.ercan@tum.de (N.E.); caterina.iuliano@googlemail.com (C.I.); dominik.cichowski12@gmail.com (D.C.); miriam.hils@tum.de (M.H.); martin.koeberle@tum.de (M.K.); 2 Global Innovation Cosmetic Ingredients, Symrise AG, 37603 Holzminden, Germany; 3 Institute of Allergology, Charite—Universitätsmedizin Berlin, Corporate Member of Freie Universität Berlin and Humboldt-Universität zu Berlin, 12203 Berlin, Germany; magda.babina@charite.de; 4 Fraunhofer Institute for Translational Medicine and Pharmacology ITMP, Immunology and Allergology, 12203 Berlin, Germany

**Keywords:** mast cells, MRGPRX2, oat extract

## Abstract

The Mas-related G protein-coupled receptor (MRGPR) X2 is expressed on skin mast cells and can be stimulated by an unusually broad spectrum of ligands, including specific drugs and even endogenous peptides. MRGPRX2 activation can induce mast cell degranulation and consequently mediator release, leading to inflammatory and hypersensitivity reactions. In addition, MRGPRX2 mediates pain and itching sensations, leading to increased efforts to identify MRGPRX2 inhibitors, including plant-derived compounds. Components within oat extracts have been shown to mediate anti-inflammatory and itch-relieving properties, but a possible inhibitory effect on MRGPRX2 activation has not yet been investigated. We aimed to fill this gap and explored whether an oat kernel extract can modulate MRGPRX2 activation. For this purpose, we established a mast cell model with the human LAD2 cell line and used it to investigate the consequences of exposure to oat extract. While we did not observe any influence on cell viability, we analyzed the impact of oat extract on MRGPRX2-mediated mast cell activation and degranulation initiated by the three confirmed MRGPRX2 ligands c48/80, substance P, and cortistatin 14. Exposure to oat extract resulted in a significant reduction in mast cell degranulation for all three ligands, as assessed by the release of β-hexosaminidase, tryptase, cell surface expression of CD63 and CD107a, and phosphorylation of ERK. All results were confirmed with primary human mast cells. Thus, we demonstrated for the first time that oat extract leads to a significant reduction in MRGPRX2 activation, pointing to a previously unrecognized capacity of natural compounds to modulate this pathway.

## 1. Introduction

Mast cells (MCs) are evolutionarily highly conserved cells that are found in almost all tissues; however, density is highest in surface organs such as the skin. Originating from hematopoietic stem cells, they migrate into tissues as precursors and, due to their high plasticity, mature in tissues in response to the local environment [1]. Through their strategic localization at tissue interfaces and in the vicinity of vessels, MCs are positioned as key sentinels in wound healing and defense against invading pathogens such as bacteria and parasites, as well as in protecting against venoms [2,3]. For this purpose, they are equipped with many mediator-filled granules in their cytoplasm, which can be rapidly released upon activation. Based on the granule composition, MCs can be categorized into two subtypes: tryptase- and chymase-expressing MCs (MC_TC_s in humans), which are predominantly found in the skin, and tryptase-only-expressing MCs (MC_T_s in humans), which are mainly found in the lung and gut.

MCs are key players in allergic inflammation, particularly in IgE-mediated type I allergic reactions, which have long been a major focus of research and clinical care. Apart from this, MCs can be stimulated to immediately release mediators via degranulation by ligands binding to the Mas-related G-protein coupled receptor X2 (MRGPRX2) [4], a member of the G protein-coupled receptor (GPCR) family that has been described to be expressed on sensory neurons, keratinocytes, basophils, and eosinophils, but it is most abundantly found on MC_TC_s in the skin [5,6]. Functionally, MRGPRX2 senses a broad range of structurally diverse secretagogues, including neuropeptides, eosinophil granule proteins, and antimicrobial peptides, as well as exogenous substances such as drugs and synthetic compounds, including quinolone antibiotics and muscle relaxants [7]. Recent three-dimensional cryo-electron microscopy of MRGPRX2 in complex with G-proteins and different ligands revealed that the structure of the active receptor differs from other GPCRs, with the presence of a wide binding domain consisting of two binding pockets that allow the receptor’s broad ligand spectrum [8,9].

Even though the mechanisms underlying MRGPRX2-mediated MC degranulation are still poorly understood, several involved downstream pathways have been identified. Upon ligand binding, a complex signaling cascade is started. While MRGPRX2 has been shown to couple to nearly all G-protein families [8,10], Gi and Gq coupling seem to be favored [11,12] and were even suggested to act synergistically in MRGPRX2-induced degranulation [13]. Next to G-protein activation, several downstream signaling pathways are involved, including β-arrestin and activation of the mitogen-activated protein (MAP) kinase pathway [11]. MAPK leads to the phosphorylation of the extracellular signal-regulated kinases 1 and 2 (ERK1/2), which has been identified as a critical mediator of degranulation, though the exact mechanisms remain unclear [12,14]. Therefore, detection of ERK1/2 phosphorylation is one marker to assess MC degranulation. Degranulation can also be quantified by measuring released mediators such as β-hexosaminidase or tryptase, a clinically used marker for MC degranulation. Furthermore, flow cytometry is used to evaluate changes in the surface exposure of CD63 and CD107a (also known as LAMP-1) since the tetraspanin CD63 and the lysosomal-associated membrane protein CD107a are MC activation markers located on intracellular granule membranes, from where they translocate to the cell surface when the cytoplasmic granules fuse with the plasma membrane during degranulation [15,16].

As a consequence of mediator release upon MRGPRX2-mediated degranulation, symptoms that are very similar to type I allergic reactions can develop and are therefore sometimes called “pseudo-allergic” reactions [4,17]. This includes local cutaneous symptoms such as erythema, efflorescence, swelling, and pruritus [18,19,20,21,22,23]. Pruritus in particular can be directly or indirectly triggered by numerous MC mediators such as histamine, tryptase, chymase, prostaglandins, leukotrienes, and certain cytokines like IL-31 and CCL2 [24,25]. Several studies further indicate that MRGPRX2 is involved in the pathogenesis of diseases characterized by chronic itching, such as chronic spontaneous urticaria [8,23,26], atopic dermatitis [27,28,29], or psoriasis [27,29], in which its expression is upregulated. Thus, recent research has focused on identifying inhibitors that can specifically modulate or suppress MRGPRX2 activation [30], including plant-derived compounds [31,32]. Oat plant (*Avena sativa* L.) ingredients have long been used in the treatment of various skin conditions, and several clinical studies have described an improvement of eczema by reduction in inflammation, transepidermal water loss, and itch after topical application of oat component-containing creams [33,34,35,36,37,38,39,40]. Key components of oats are avenanthramides (Avns), a group of bioactive phenolic compounds, with at least 40 naturally occurring Avns being identified so far. They vary in the combination and substitution pattern of the phenylalkylenoic acid (cinnamic acid or avenalumic acid) and anthranilic acid moiety [41]. Among these, the three typically most abundant Avns, Avn A, B, and C, have been widely studied [42]. In recent years, Avns have attracted increasing scientific interest as they have been shown to exhibit anti-inflammatory and anti-itch activity via the inhibition of the NF-kB pathway [43] and to improve skin barrier function by upregulation of tight junction expression [44] in keratinocytes. Furthermore, Dhakal et al. demonstrated that Avn C inhibits IgE-mediated release of the inflammatory mediators IL-4, IL-6, and TNF-α from MCs and a dose-dependent attenuation of anaphylactic reactions in a preclinical model [42]. However, to date, no studies have investigated the influence of oat compounds on MRGPRX2 activity.

In the current study, we aimed to explore whether *Avena sativa* (oat) kernel extract acts as a modulator of MRGPRX2 activation. For this purpose, we established a mast cell model with the Laboratory of Allergic Diseases (LAD)2 cells, a widely used human MC line that originates from bone marrow aspirates of a patient with mastocytosis [45]. We mainly focused on three different MRGPRX2 agonists with confirmed receptor binding by cryo-electron microscopy: the synthetic polymer compound 48/80 (c48/80) and the two endogenous ligands substance P (S.P.) and cortistatin 14 (CST-14) [8]. c48/80 is a powerful MRGPRX2 ligand that induces MC degranulation and is considered the gold standard for in vitro studies [14]. S.P. and CST-14 are neurotransmitters implicated in contributing to the itch associated with atopic dermatitis and chronic spontaneous urticaria, but also in skin with pruritus in the absence of visible signs of disease [25,46,47,48,49].

We demonstrate that exposure of immortalized and primary mast cells to oat extract results in a significant reduction in MRGPRX2-mediated activation, pointing to a previously unrecognized capacity of natural compounds to modulate this pathway.

## 2. Results

### 2.1. MRGPRX2 Expression and Activation in Human Mast Cell Line LAD2

**Figure 1:**
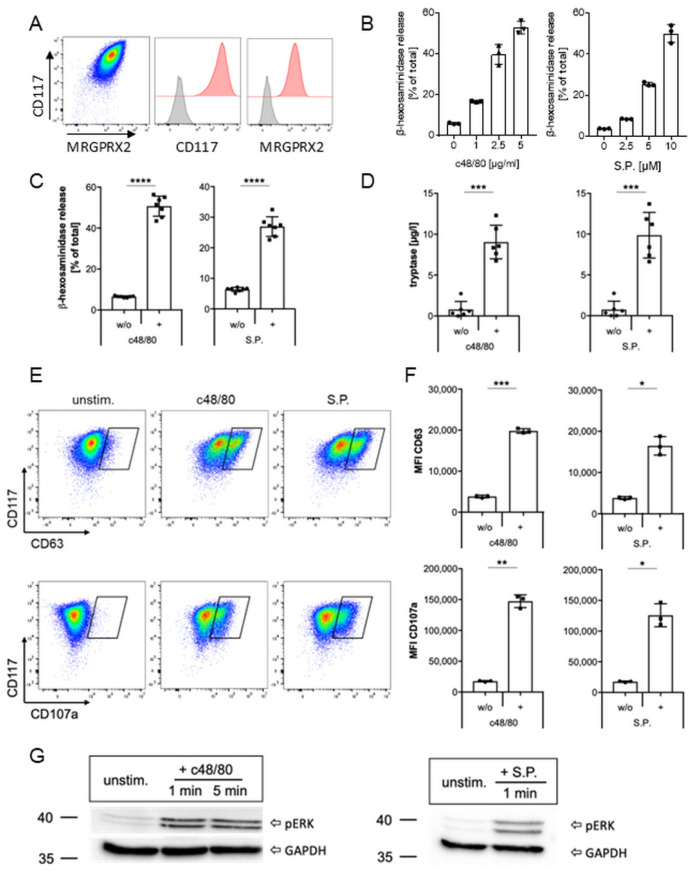
MRGPRX2 expression and activation in human mast cell line LAD2. (**A**) Representative dot plot and histograms showing the expression of MC receptors CD117 and MRGPRX2 (shown in red in each case) in LAD2 cells. Isotype control is shown in grey. (**B**) Dose-dependent β-hexosaminidase release upon stimulation of LAD2 cells with different MRGPRX2 ligands (one of two experiments, each with triplicates). (**C**–**G**) LAD2 were stimulated with 2.5 µg/mL c48/80 or 5 µM S.P., and degranulation was assessed compared to unstimulated cells by β-hexosaminidase (**C**), tryptase (**D**), CD63, and CD107a exposure shown as representative dot plots (**E**) or quantification of MFI (**F**), and ERK-phosphorylation by Western blot (one of two experiments). A summary of 7 (**C**) or 6 (**D**,**F**), experiments is shown. * *p* < 0.05, ** *p* < 0.01, *** *p* < 0.001, **** *p* < 0.0001.

LAD2 cells are known to express MRGPRX2 and to degranulate upon activation of this receptor [50]. To establish a model for investigating MRGPRX2 modulation with this MC line, we first examined the expression of MRGPRX2 on LAD2 cells and detected high levels of the receptor as well as the stem cell factor receptor CD117 on their surface (Figure 1A). With two of the most extensively studied balanced MRGPRX2 ligands—the synthetic compound 48/80 (c48/80) and the neuropeptide substance P (S.P.) we confirmed that both ligands activated LAD2 cells in a dose-dependent manner by means of a β-hexosaminidase assay (Figure 1B). Similar results were obtained with the neuropeptide cortistatin-14 (CST-14), another balanced ligand of MRGPRX2, which has been demonstrated to be linked to the pathogenesis of chronic prurigo [46] (Supplementary Figure S1). Based on this outcome, LAD2 cells were subsequently stimulated with 2.5 µg/mL c48/80, 5 µM S.P., or 5 µM CST-14, as this resulted in a robust release of approximately 50%, 30%, or 40% of total β-hexosaminidase, respectively, (Figure 1C, Supplementary Figure S1B). Activation-induced degranulation of LAD2 cells was further demonstrated by a significant elevation in the secretion of tryptase, a clinically relevant marker for MC degranulation in the serum (Figure 1D, Supplementary Figure S1C); a rise in CD63 and CD107a on the MC surface assessed by flow cytometry (Figure 1E,F, Supplementary Figure S1D), and an increase in phosphorylated ERK as demonstrated by Western blot (Figure 1G).

### 2.2. Inhibition of MRGPRX2 Activation in LAD2 Cells

**Figure 2:**
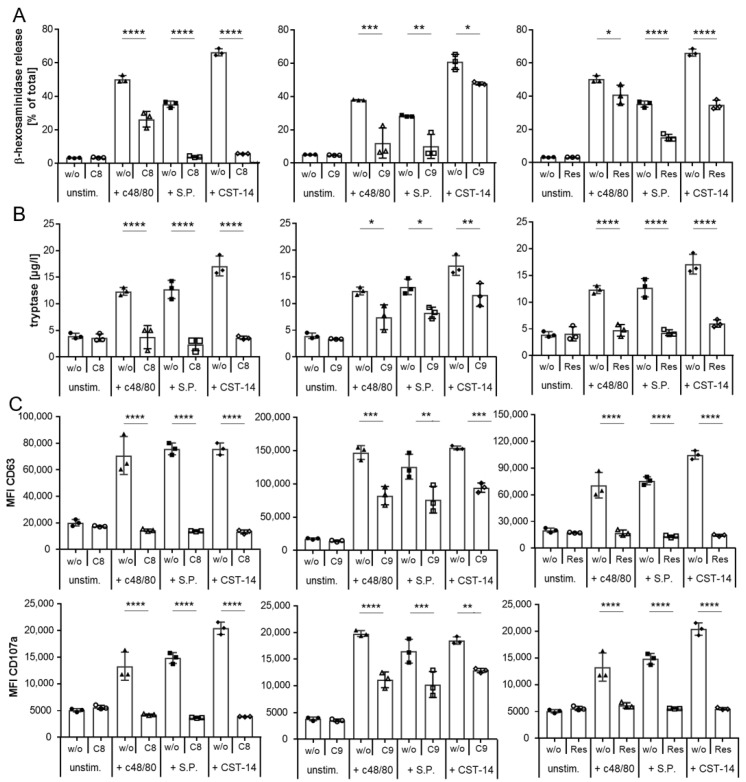
Inhibition of MRGPRX2 activation by known antagonists. LAD2 cells were stimulated with c48/80, S.P., or CST-14 with or without exposure to the known inhibitors C8 (**left** side), C9 (**middle**), or resveratrol (**right**) side). Inhibition of MRGPRX2 was assessed by β-hexosaminidase release (**A**), tryptase release (**B**), and changes in surface expression of CD63 ((**C**), upper panel) and CD107a ((**C**), lower panel). A representative experiment of two is shown for (**A**–**C**), each with triplicates. * *p* < 0.05, ** *p* < 0.01, *** *p* < 0.001, **** *p* < 0.0001.

Next, we examined whether we could inhibit MRGPRX2 activation with known inhibitors using these settings. To this end, we have selected three different defined inhibitors of MRGPRX2 activation: the small molecule antagonists C8 and C9 [51] and the natural polyphenolic compound resveratrol [52]. After defining the appropriate concentrations of these antagonists (Supplementary Figure S2), their impact on LAD2 degranulation mediated by MRGPRX2 activation was investigated. As depicted in Figure 2, we could demonstrate a significant inhibition of degranulation for all three antagonists as assessed by β-hexosaminidase (Figure 2A) and tryptase (Figure 2B) release, as well as by reduced expression of the MC activation surface markers CD63 and CD107a (Figure 2C) induced by c48/80, S.P., or CST-14. In all cases, all three detection methods showed a significant reduction in degranulation, with C8 and resveratrol proving more effective than C9, particularly in MRGPRX2 activation by S.P. and CST-14 (Figure 2). Thus, our LAD2 model is suitable to investigate the inhibition of MRGPRX2 activation as shown by the three representative agonists.

### 2.3. Evaluation of Oat Extract as a New Inhibitor of MRGPRX2 Activation with LAD2 Cells

**Figure 3:**
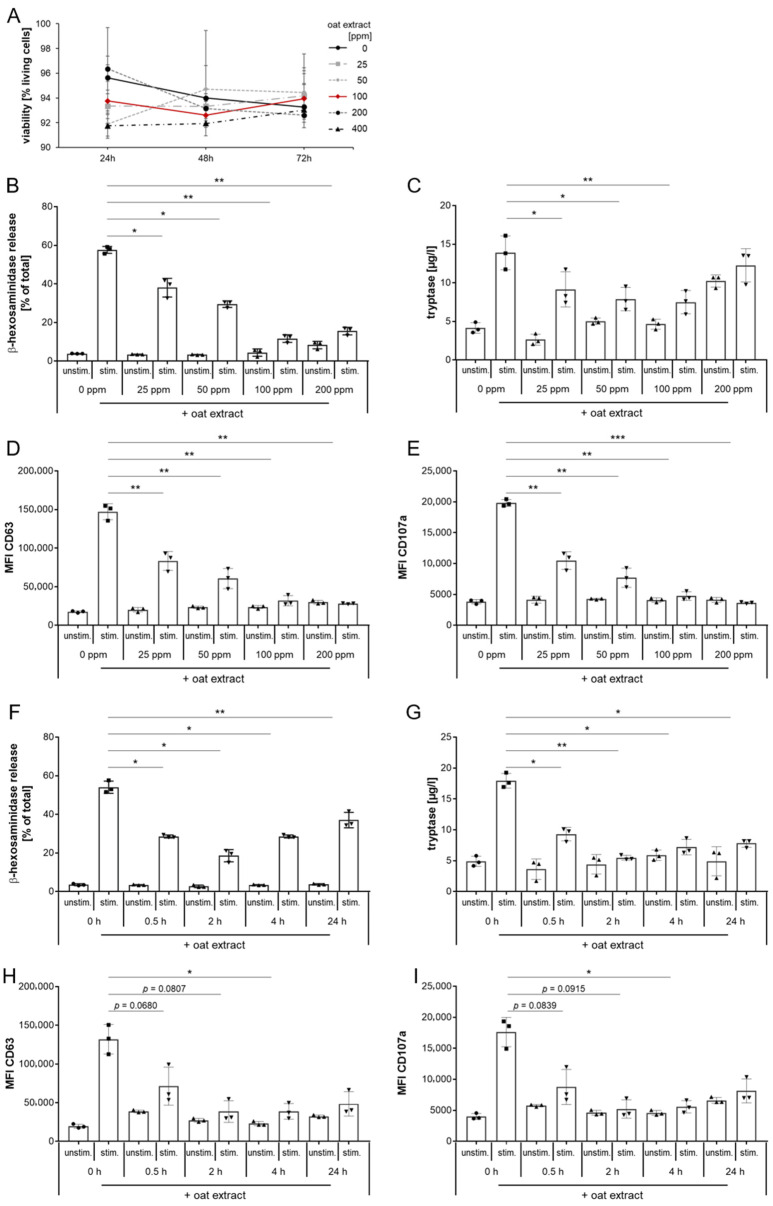
Evaluation of oat extract as inhibitor of MRGPRX2. (**A**) LAD2 cells were exposed to increasing doses of oat extract over 72 h. Viability was recorded every 24 h by counting live and dead cells. LAD2 cells were exposed to different doses of oat extract for 2 h (**B**–**E**) or to 100 ppm oat extract for different time periods (**F**–**I**) before stimulation with c48/80. LAD2 activation was assessed by β-hexosaminidase release (**B**,**F**), tryptase release (**C**,**G**), and changes in surface expression of CD63 (**D**,**H**) and CD107a (**E**,**I**). One representative experiment of two (**A**) or three (**B**–**I**), each with triplicates, is shown. * *p* < 0.05, ** *p* < 0.01, *** *p* < 0.001.

After confirming both the induction and inhibition of MRGPRX2-mediated degranulation in our LAD2 model with known agonists and antagonists, we wanted to investigate the effect of a novel oat kernel extract as a potential new modulator of MRGPRX2. We first analyzed whether the extract has cytotoxic potential against MCs. For this purpose, we incubated LAD2 cells with varying amounts of oat extract for 24, 48, and 72 h and determined the cell viability. As shown in Figure 3A, the addition of oat extract in the concentrations used did not impact the viability of cultured LAD2 cells, with no significant difference in the amount of living cells between MCs cultured with or without (solid black line) the extract.

Based on these findings, we next analyzed the consequences of the oat extract on the MRGPRX2 activation via c48/80. We pre-exposed LAD2 cells to different concentrations of the oat extract indicated in Figure 3A. β-hexosaminidase release assay revealed a significant reduction in c48/80-induced degranulation for all concentrations used, whereby 100 ppm oat extract proved to be the most effective (Figure 3B). This was further confirmed by measurement of tryptase release (Figure 3C) and the cell surface expression of CD63 and CD107a (Figure 3D,E), all of which showed the most effective reduction in degranulation by 100 ppm oat extract. Therefore, we decided to use this concentration in the subsequent experiments. In the next step, we investigated the impact of exposure duration of the extract on MRGPRX2 activation. To this end, we incubated LAD2 cells for different time periods with 100 ppm oat extract before stimulating them with c48/80. As shown by β-hexosaminidase release, all selected exposure times led to a significant reduction in MRGPRX2 activation, resulting in reduced cell degranulation (Figure 3F). The decrease in degranulation was most pronounced after 2 h of exposure with 100 ppm oat extract, as demonstrated by β-hexosaminidase (Figure 3F), tryptase (Figure 3G), and CD63 and CD107a determination (Figure 3H,I). Based on these results, we continued in the following experiments using 2 h exposure to 100 ppm oat extract.

### 2.4. Inhibition of MRGPRX2 Activation in Human LAD2 Mast Cells by Oat Extract

**Figure 4:**
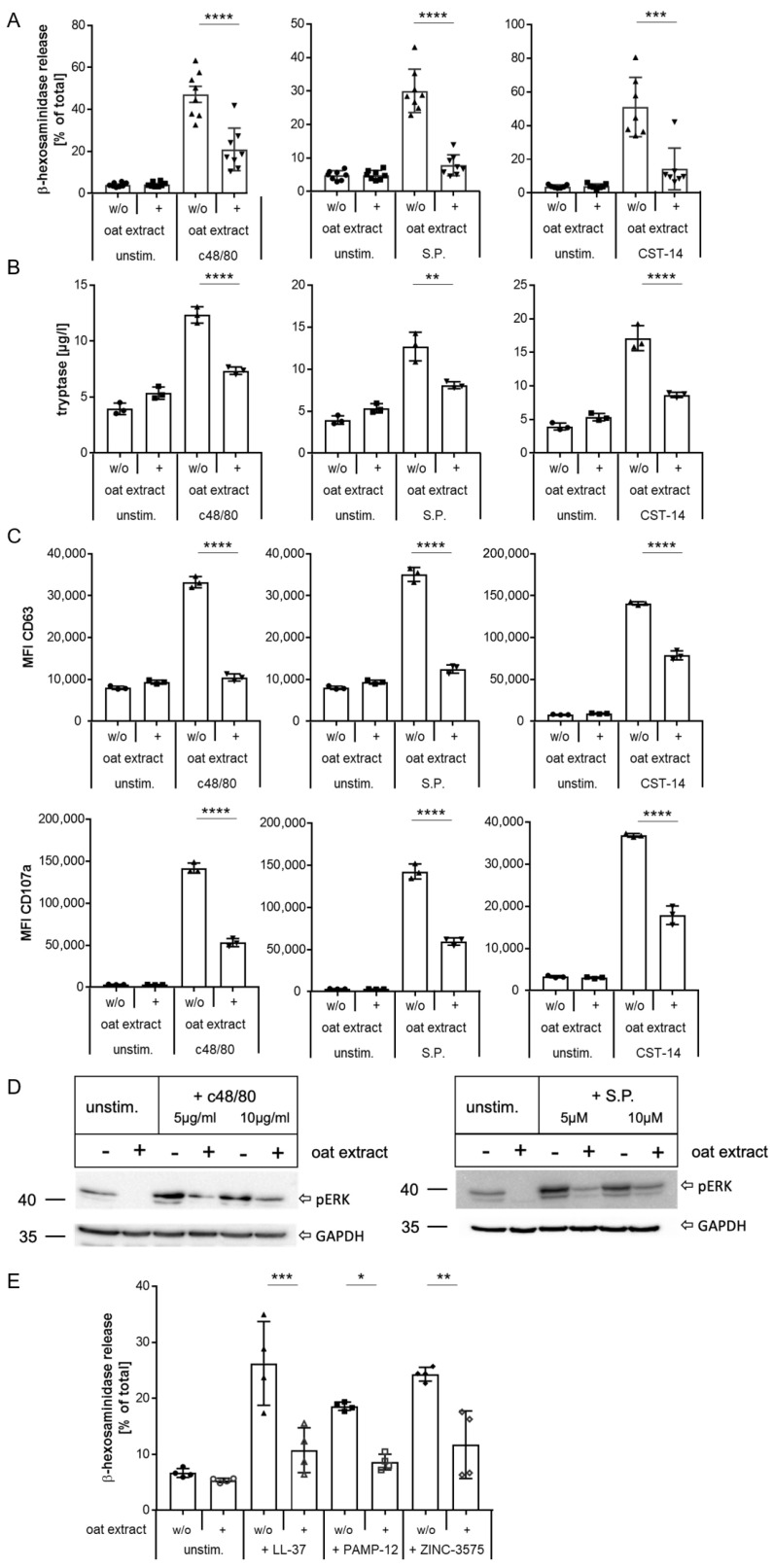
Inhibition of MRGPRX2 activation in human mast cell line LAD2 by oat extract. β-hexosaminidase release (**A**) tryptase release ((**B**) and changes in surface expression of CD63 and CD107a (**C**) after stimulation of LAD2 cells with c48/80, S.P., and CST-14 with or without pre-exposure to oat extract. (**D**) Western blot showing reduction in ERK-phosphorylation (pERK) of stimulated LAD2 cells after pre-exposure to oat extract (one of two experiments is shown in each case). GAPDH was used as loading control. (**E**) β-hexosaminidase release from LAD2 cells after stimulation with MRGPRX2 ligands LL37, PAMP-12, and ZINC-3575 with and without oat extract pre-exposure. (**A**): summary of 7–8 experiments, each performed in duplicates or triplicates. (**B**,**C**): one representative experiment with triplicates out of 5–6 experiments; (**D**): one representative experiment of two; (**E**): summary of 2 experiments with duplicates. * *p* < 0.05, ** *p* < 0.01, *** *p* < 0.001, **** *p* < 0.0001.

Having established these conditions, we first examined the modulation of MRGPRX2 activation by oat extract in detail in LAD2 cells. As shown in Figure 4, exposure to oat extract led to a significant reduction in activation and subsequent degranulation of the cells with all three MRGPRX2 ligands, demonstrated by reduced release of β-hexosaminidase and tryptase (Figure 4A,B, respectively) and less translocation of the activation markers CD63 and CD107a to the cell surface (Figure 4C).

Additionally, we examined the phosphorylation of ERK as an early consequence of MRGPRX2 activation. As shown by Western blot, the exposure to oat extract clearly reduces the induction of ERK-phosphorylation mediated by MRGPRX2 activation (Figure 4D). Finally, we investigated the impact of oat extract on the stimulation of MRGPRX2 via three additional agonists with different signaling characteristics: the antimicrobial peptide LL37, a G-protein-biased MRGPRX2 ligand, proadrenomedullin peptide N-terminal 12 (PAMP-12) [53], and the small molecule ZINC-3575 [54]. Exposure to oat extract significantly reduced MRGPRX2 activation evoked by all three agonists (Figure 4E). These data demonstrate that pre-exposure to oat extract modulates the responsiveness of the MRGPRX2 to balanced and G-protein-biased agonists, resulting in a significant reduction in degranulation of LAD2 cells.

LAD2 cells have been shown to exhibit an aberrant β-arrestin system leading to hyperresponsiveness [55]. We therefore performed a PathHunter^®^β-Arrestin assay that monitored the impact of oat extract on the recruitment of β-arrestin upon MRGPRX2 activation in CHO cells transfected with a modified version of MRGPRX2. As with LAD2 cells, oat extract efficiently inhibited MRGPRX2 activation in this system (Supplementary Figure S3), confirming that the observed modulation of MRGPRX2-induced degranulation was not LAD2-specific.

### 2.5. Inhibition of MRGPRX2 Activation in Primary Human Mast Cells by Oat Extract

**Figure 5:**
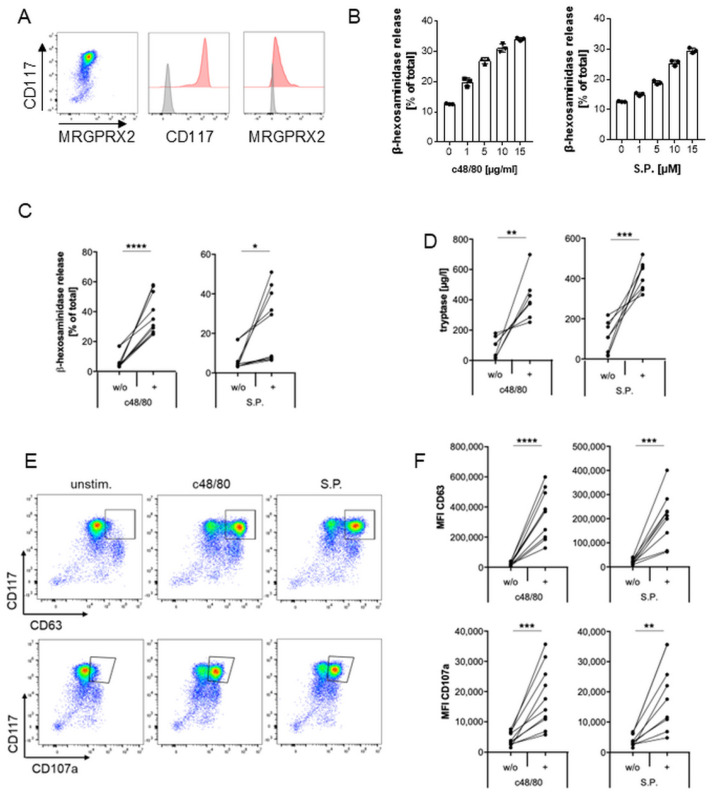
Activation of MRGPRX2 in primary human skin mast cells. (**A**) Dot plot and histograms showing the expression of MC receptors CD117 and MRGPRX2 (shown in red in each case) in skin MCs. Isotype control is depicted in grey. (**B**) Dose-dependent β-hexosaminidase release upon stimulation with different MRGPRX2 ligands (representative example out of two performed with triplicates from one donor pool). (**C**–**F**) skin MCs were stimulated with 5 µg/mL c48/80 or 10 µM S.P., and degranulation was assessed compared to unstimulated cells by β-hexosaminidase ((**C**), *n* = 10), tryptase ((**D**), *n* = 7–8), and CD63 and CD107a expression depicted as dot plots ((**E**), representative example of one donor pool), or MFI ((**F**), *n* = 9–10). (**C**,**D**,**F**): Each data point represents a donor pool and is based on a single value or the average of duplicates. “*n*” indicates the number of donor pools analyzed. * *p* < 0.05, ** *p* < 0.01, *** *p* < 0.001, **** *p* < 0.0001.

Subsequently, we wanted to validate our findings with primary MCs. Blood-derived (BD) MCs are a common tool for ex vivo MC studies with higher physiological relevance compared to MC cell lines. Given the high plasticity of MCs influenced by the surrounding tissue environment, skin MCs probably serve as the best cell culture model for MRGPRX2-induced responses in vivo. Following the setup in LAD2 cells, we thus first confirmed MRGPRX2 expression in skin MCs and BDMCs (Figure 5A; Supplementary Figure S4A, respectively), and their response to MRGPRX2 ligands (Figure 5B–F, Supplementary Figure S4B–E). Compared to LAD2 cells, we found a significantly higher release of tryptase from primary MCs upon MRGPRX2 activation (Figure 5D, Supplementary Figure S4C), which is in line with published data [56]. Additionally, we observed donor-dependent variations in the response to the ligands for the four degranulation markers assessed: β-hexosaminidase, tryptase, median fluorescence intensity (MFI) of CD63, and MFI of CD107a (Figure 5, Supplementary Figure S4).

**Figure 6:**
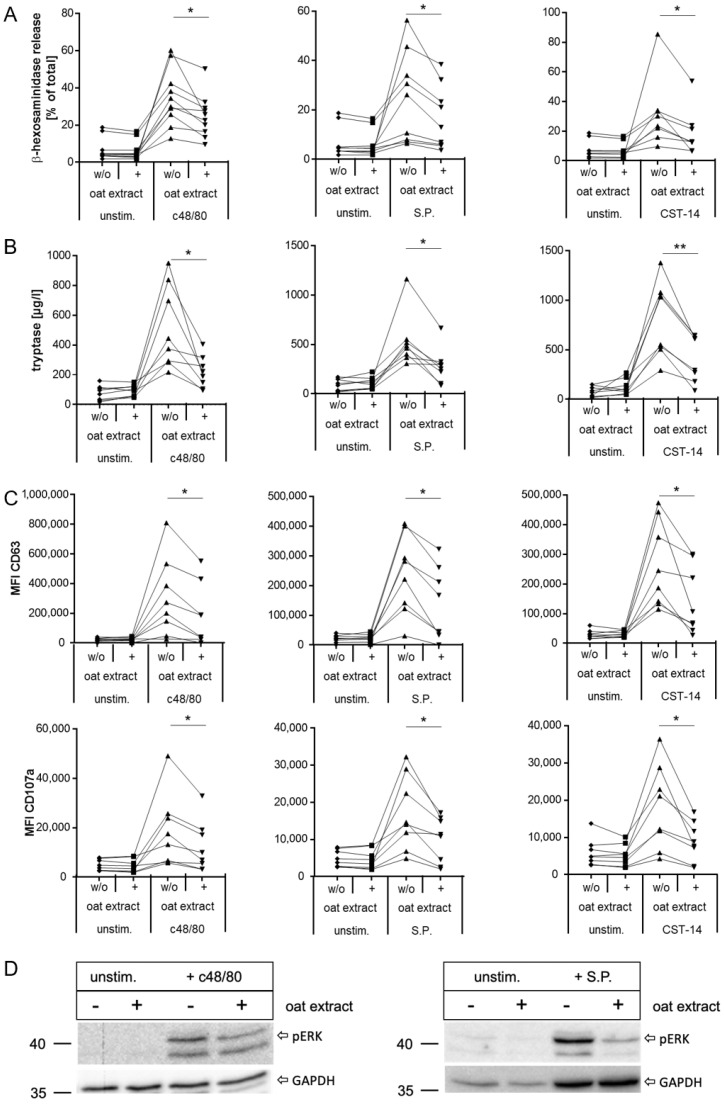
Inhibition of MRGPRX2 activation in primary human mast cells by oat extract. Skin MCs (**A**–**C**) or BDMCs (**D**) were exposed to oat extract followed by stimulation with different MRGPRX2 ligands, and activation was assessed by different means. (**A**) β-hexosaminidase release (*n* = 8–10), (**B**) tryptase release (*n* = 8); (**C**) changes in surface expression of CD63 and CD107a (*n* = 8). (**D**) Western blot showing pERK in BDMCs with and without exposure to oat extract before stimulation with c48/80 (**left**) or S.P. (**right**) (one representative experiment of two). (**A**–**C**): each datapoint represents one donor pool as single value or the average of duplicates. “*n*” indicates the number of donor pools analyzed. * *p* < 0.05, ** *p* < 0.01.

In the next step, we investigated the effect of oat extract on MRGPRX2 activation in primary cells. As in LAD2 cells, in primary MCs, ligand-induced degranulation was significantly reduced upon pre-exposure to oat extract, resulting in decreased release of β-hexosaminidase (Figure 6A, Supplementary Figure S5A).

This was also reflected in the decrease in tryptase release (Figure 6B, Supplementary Figure S5B) and the reduction in surface exposure of CD63 and CD107a (Figure 6C, Supplementary Figure S5C). Western blot analysis also confirmed reduced activation of signal transduction after MRGPRX2 activation with oat extract pre-exposure, as shown by reduced levels of phosphorylated ERK (Figure 6D). Compared to LAD2, donor-dependent variability was evident in primary MCs, as already observed for stimulation (Figure 5). We have thus shown that oat extract can significantly inhibit MRGPRX2-mediated degranulation in both the human cell line LAD2 and primary human MCs as assessed by the release of β-hexosaminidase and tryptase, the increase in CD63 and CD107a on the mast cell surface, and the phosphorylation of ERK1/2.

### 2.6. Oat Extract Inhibits the MRGPRX2-Induced Synthesis of CCL2 in LAD2 Cells and Primary Human Mast Cells

**Figure 7:**
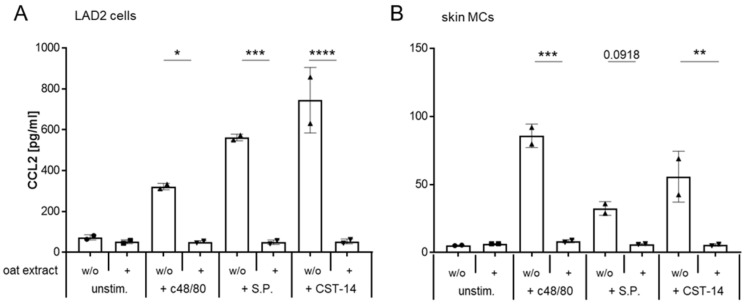
Inhibition of newly synthesized chemokine CCL2 by oat extract. LAD2 cells (**A**) and skin MCs (**B**) were exposed to oat extract before stimulation with c48/80, S.P., or CST-14. After 24 h, CCL2 was quantified in the cell culture supernatant by a bead-based ELISA. One of two experiments with duplicates is shown. * *p* < 0.05, ** *p* < 0.01, *** *p* < 0.001, **** *p* < 0.0001.

As a final step, we asked whether the oat extract also influences the release of newly synthesized mediators. To investigate this, we exposed LAD2 cells and skin MCs to oat extract for 2 h, followed by stimulation with the three MRGPRX2 ligands. 24 h later, we quantified the amount of the chemokine CCL2 in the cell culture supernatant. CCL2, also known as monocyte chemoattractant protein (MCP-1), has been shown to be released upon MRGPRX2 activation by S.P. [57]. CCL2 is a potent chemoattractant that recruits several immune cells to sites of release [58] and has been described to be involved in the elicitation of itch via the activation of its receptor CCR2 [24,59]. As shown in Figure 7, the release of CCL2 induced by c48/80, S.P., and CST-14 was completely inhibited after pre-exposure to oat extract in LAD2 cells (Figure 7A) as well as in skin MCs (Figure 7B). These results show that the oat extract not only has a short-term effect on the release of preformed mediators but also inhibits the MRGPRX2-induced re-synthesis of active substances.

In summary, our data demonstrate that exposure to oat extract modulates MRGPRX2 activation with regard to both immediate and delayed MC response, resulting in a significant reduction in mediator release. Given that these mediators contribute to itching, our data indicate that this oat extract might be used to alleviate or prevent pruritus.

## 3. Discussion

Sensitive skin is a prevalent concern worldwide, and its incidence continues to rise. It encompasses a spectrum of conditions, including reactive skin, characterized by susceptibility to irritants and manifestations such as erythema, pruritus, burning, or stinging sensations. Therefore, the care for such skin conditions requires ingredients relieving these symptoms. In order to meet these needs, a deep understanding of the mechanisms driving hypersensitivity is necessary.

Since its identification as a receptor that mediates IgE-independent activation of MCs, interest in MRGPRX2 and its signaling pathways has been growing, which has contributed greatly to a better understanding of the previously unresolved issue of hypersensitivity and pseudo-allergic reactions [17]. In the course of the associated research, it has been shown that MRGPRX2 plays a crucial role in triggering pruritus, which makes the receptor an attractive target for the management of itching. However, many questions regarding the elicitation and regulation of the MRGPRX2 signaling cascade remain to be resolved. First, there is the very broad range of ligands differing in size, charge, and origin. Cryo-electron microscopy has unraveled an unusual binding domain that is divided into a negatively charged pocket, which seems to trigger the receptor activation, and a hydrophobic pocket [8,9]. The negatively charged pocket is very small and can only accommodate a single chain of a residue. This paucity of a specific binding site allows many basic peptides to bind and thereby activate MRGPRX2, explaining the receptor’s broad ligand spectrum [8]. However, the exact crystal structure of MRGPRX2 is missing. In view of this, the complex downstream pathway is also not yet fully understood. Cao et al. have discovered a promiscuous signaling profile for MRGPRX2, as the receptor can activate all four G protein subfamilies [8], and both the inhibitory Gi- and the activating Gq-coupled pathways are activated [8,50,54] and even seem to act synergistically [13]. Beyond the activation of these G protein-associated signaling pathways, ligand-specific intracellular modulations can occur. So-called balanced ligands can additionally initiate β-arrestin-mediated receptor internalization, desensitization, and signal termination [60]. Balanced ligands include c48/80, S.P., CST-14, PAMP-12 [50,61,62,63], and ZINC-3575 [54]. In contrast, biased MRGPRX2 ligands, e.g., LL37 [22], do not recruit β-arrestin, indicating a lack of receptor internalization and desensitization [11,30,53,54,62].

Furthermore, as also observed in our experiments, a high donor-dependent variability in response to the same agonists has been reported for primary human mast cells as well as for skin explants. One contributing factor is the high degree of expression heterogeneity with an inter-donor variability described for BDMCs [64] and MCs isolated from human explants [15]. In this context, MRGPRX2 expression, but also receptor responsiveness, can be influenced by microenvironmental factors like cytokines [65,66,67] and cell conditions [66,68]. Additionally, MRGPRX2 has been reported to be highly polymorphic with many naturally occurring mutations, including loss-of-function alterations leading to receptor unresponsiveness [69,70] as well as gain-of-function mutations resulting in hyperresponsiveness [71,72]. Interestingly, enhanced expression of MRGPRX2 as well as gain-of-function mutations have been associated with diseases linked to pruritus [47].

Itching, especially in its chronic form, can have a significant impact on quality of life, and many conditions associated with itching, such as AD, are on the rise worldwide. For skin prone to itching, it is recommended to use moisturizing and lipid-replenishing creams. In clinical studies, oat components added to creams have been shown to have anti-inflammatory and anti-itching effects, although the mechanism of action is not fully understood. Given the new findings on MRGPRX2, it is reasonable to assume that this effect is at least partly mediated by the modulation of receptor activation on MCs. However, no studies have been conducted on this topic to date. With our MC model, we provide the MRGPRX2 receptor and its induced signaling as a novel target to treat itch in hypersensitivity and atopic-prone skin. We were able to show for the first time that oat extract does indeed modulate the responsiveness of MRGPRX2 to its ligands, thereby attenuating the signaling cascades involved. The study suggests that the oat extract has significant potential as an inhibitor of mast cell activation. It consistently modulates MRGPRX2-induced signaling, diminishes ERK phosphorylation, and reduces degranulation. These findings support further investigation into the use of the oat extract as a therapeutic agent for mast cell-related disorders, including pruritus. Furthermore, our MC model allows the identification and characterization of new ingredients that inhibit MRGPRX2-induced itch sensation.

### Limitations of the Study

While we clearly demonstrated an inhibitory effect of oat extract on MRGPRX2-mediated mast cell activation, we have not addressed which components of the extract are responsible for the inhibition and what needs to be investigated in more detail. Furthermore, the exact molecular mechanism by which MRGPRX2 is inhibited also remains to be deciphered, e.g., whether the inhibition occurs by blocking the binding sites of the MRGPRX2 or whether the extract or components thereof translocate into the interior of the mast cells and exert an inhibitory effect there, e.g., on the downstream signaling pathways. This should be the subject of further studies.

## 4. Materials and Methods

### 4.1. Cell Culture and Reagents

Mast cells (MCs) were maintained at 37 °C and 5% CO_2_, adjusted to 0.5–1 × 10^6^ cells/mL with fresh medium, recombinant human stem cell factor (rhSCF; Miltenyi Biotec, Bergisch Gladbach, Germany) at 100 ng/mL for LAD2 and skin-derived mast cells and 125 ng/mL for blood-derived mast cells, and cytokines replenished twice per week along with cell viability assessment by Trypan blue (Carl ROTH, Karlsruhe, Germany). All cultured media were tested regularly for mycoplasma, and no contamination has been detected.

LAD2 cells (Applied Biological Materials, Richmond, BC, Canada) were cultured in StemPro-34 serum-free medium (Thermo Fisher Scientific, Darmstadt, Germany) supplemented with 2.5% StemPro-34 supplements, 1% penicillin/streptomycin (P/S; 100 U/mL/100 µg/mL; Merck, Darmstadt, Germany), and 1% 2 mM L-glutamine (Merck, Germany). Medium without StemPro-34 supplement was used for starvation.

Blood-derived MCs were generated in vitro from CD34^+^ progenitor cells in peripheral human blood as previously described [73]. Briefly, PBMCs were obtained from buffy coat concentrates of healthy donors (Bayerisches Rotes Kreuz; München, Germany) using gradient centrifugation with Biocoll (Merck, Darmstadt, Germany), and CD34^+^ cells were selected using the EasySep Human CD34 Positive Selection Kit II (STEMCELL Technologies, Köln, Germany) according to the manufacturer’s instructions. Isolated CD34^+^ cells were cultured in StemSpan SFEM (STEMCELL Technologies, Köln, Germany) with 10% expansion supplement and 1% P/S for one week, followed by culture in StemSpan SFEM containing 1% P/S, rhSCF (Miltenyi Biotec, Bergisch Gladbach, Germany), and from week 2 with 20 ng/mL recombinant human interleukin-3 (rhIL3) (BioLegend, Koblenz, Germany). After culture, upon achieving ≥70% CD117^+^ cells, the culture was further enriched using CD117 MicroBeads and QuadroMACS Separator (Miltenyi Biotec, Bergisch Gladbach, Germany). Enriched cells were subsequently maintained in StemSpan SFEM supplemented with 1% P/S, rhSCF, and 50 ng/mL rhIL-6 (R&D Systems, Wiesbaden, Germany). Enrichment efficiency was confirmed by flow cytometry for CD117 and FcεRI (BioLegend, Koblenz, Germany) expression. For stimulation, Dulbecco’s Modified Eagle Medium GlutaMAX high glucose medium (Thermo Fisher Scientific, Darmstadt, Germany) supplemented with 1% HEPES (Merck, Darmstadt, Germany), 1% MEMAA, 1% P/S, and 0.1% β-mercaptoethanol (Thermo Fisher Scientific, Darmstadt, Germany) was used.

Skin MCs were isolated from foreskin from circumcisions of healthy human donors as previously described [61,74] and cultured and expanded in Iscove’s Basal Medium (Merck, Darmstadt, Germany) supplemented with 10% fetal bovine serum (FBS; Gibco, Darmstadt, Germany), 1% P/S, 1% minimal essential medium amino acids (Merck, Germany), 0.002% 1-thioglycerol (GE Healthcare, Freiburg, Germany), rhSCF, and 20 ng/mL rh IL-4 (Thermo Fisher Scientific, Darmstadt, Germany). The starvation medium did not contain FBS. Due to the low yield, pools with MCs from several donors were used for the experiments.

### 4.2. MC Stimulation

16–18 h prior to stimulation, 1 × 10^5^/mL MCs were seeded into cell-type-specific starvation medium. If not otherwise stated, MCs were stimulated with the MRGPRX2 agonists c48/80 (Merck, Darmstadt, Germany; 2.5 µg/mL for LAD2, 5 µg/mL for primary MCs), substance P (Merck, Darmstadt, Germany; 5µM for LAD2, 10 µM for primary MCs), or 5 µM cortistatin-14 (R&D Systems, Wiebaden, Germany). Stimulation was carried out for 20 min in Tyrode’s buffer (Merck, Darmstadt, Germany) when measuring β-hexosaminidase release and for 5 min in specific starvation medium when assessing activation marker expression by flow cytometry.

*Avena sativa* (oat) kernel extract, containing 2.4% of avenanthramides A, B, and C in sum and produced via a patented technology, was provided by Symrise AG (Holzminden, Germany), and 1000 ppm oat extract was dissolved in 1% DMSO (Merck, Darmstadt, Germany). Unless otherwise specified, MCs were pre-exposed to 100 ppm oat extract for 2 h in starvation medium prior to stimulation with the agonists.

In selected experiments, cells were exposed to additional MRGPRX2 inhibitors C8 (MedChemExpress via Biozol, Hamburg, Germany), C9 (Merck, Darmstadt, Germany), or trans-resveratrol (Merck, Darmstadt, Germany) in the corresponding starvation medium for 20 min. Variations in agonist or antagonist concentration or incubation time are specified in the corresponding figure legends.

### 4.3. β-Hexosaminidase Assay

β-Hexosaminidase release upon stimulation was measured as previously described [75]. In experiments involving oat extract pre-treatment, cells were washed three times with Tyrode’s buffer prior to stimulation to prevent autofluorescence from oat extract interfering with absorbance measurements.

### 4.4. Flow Cytometry Analysis and Tryptase Measurement

Cells were centrifuged immediately after stimulation with agonists, and the supernatants were collected. Cells were fixed with intracellular fixation buffer (Thermo Fisher Scientific, Darmstadt, Germany) for 30 min, blocked with FcR blocking reagent (Thermo Fisher Scientific, Darmstadt, Germany), and stained with fluorochrome-conjugated antibodies against CD117, FcεRI, CD107a, MRGPRX2, and CD63 (BioLegend, Koblenz, Germany). Data were acquired on a CytoFLEX flow cytometer (Beckman Coulter, Krefeld, Germany). Supernatants were assayed for released tryptase using the ImmunoCAP Tryptase assay on a Phadia 250 (Thermo Fisher Scientific, Darmstadt, Germany) according to the manufacturer’s instructions.

### 4.5. CCL2 Assay

After pre-exposure to oat extract, MCs were stimulated with different agonists for 24 h. Supernatants were measured using human LEGENDplex (BioLegend, Koblenz, Germany), and data were acquired using BD FACS Canto (BD Biosciences, Heidelberg, Germany).

### 4.6. Western Blot

Following stimulation, cells were lysed in RIPA buffer (Thermo Fisher Scientific, Darmstadt, Germany) supplemented with 1% Halt protease and phosphatase inhibitor cocktail (Thermo Fisher Scientific, Darmstadt, Germany) and incubated on ice for 15 min. Lysates were centrifuged at 14,000× *g* for 15 min at 4 °C, and protein concentrations were determined using the BCA protein assay kit (Thermo Fisher Scientific, Darmstadt, Germany). Equal amounts of protein were separated by SDS-PAGE, transferred to nitrocellulose membranes (Bio-Rad Laboratories, Feldkirchen, Germany), and probed with primary antibodies against phospho-ERK1/2 and GAPDH (Thermo Fisher Scientific, Darmstadt, Germany). Horseradish peroxidase–conjugated anti-rabbit IgG (Cell Signaling Technology, Frankfurt a. Main, Germany) was used as the secondary antibody for detection.

### 4.7. Statistical Analysis

The data were processed via GraphPad Prism 7.05 (GraphPad Software via Statcon, Göttingen, Germany). Flow cytometry analysis was performed with FlowJo_V10 (BD Bioscience, Heidelberg, Germany), and LEGENDplex data were analyzed using LEGENDplex Data Analysis Software Suite (version 8). Each experiment was performed at least twice. The experimental results are expressed as the means ± SDs. Comparisons were performed via unpaired Student’s t-test and one-way analysis of variance (ANOVA) and Tukey’s multiple-comparisons test if not otherwise stated. For all analyses, a two-tailed *p*-value < 0.05 was considered statistically significant.

## Data Availability

The original contributions presented in this study are included in the article/Supplementary Material. Further inquiries can be directed to the corresponding author(s).

## Supplementary Materials

The following supporting information can be downloaded at: https://www.mdpi.com/article/10.3390/ijms27010334/s1.

## Abbreviations

The following abbreviations are used in this manuscript:

MC	Mast Cell
MRGPRX2	Mas-related G-protein coupled receptor X2
MC_T_	Tryptase expressing MCs
MC_TC_	Tryptase- and chymase expressing MCs
FcεRI	Fcε receptor I
GPCR	G-protein coupled receptor
Avn	Avenanthramide
oat	*Avena sativa*
LAD2	Laboratory of allergic disease 2
CST-14	Cortistatin 14
S.P.	Substance P
c48/80	Compound 48/80
MFI	Mean fluorescence intensity
Res	resveratrol
