# Supplementary material for: Modulation of Mast Cell Activation via MRGPRX2 by Natural Oat Extract
Source: Int J Mol Sci. 2025 Dec 28;27(1):334. doi: 10.3390/ijms27010334 (PMC12786182; doi:10.3390/ijms27010334)
Supplement: Supplementary file 1 [file ijms-27-00334-s001.zip › ijms-4046532-supplementary.pdf]

Supplementary Material

Modulation of mast activation via MRGPRX2 by natural oat extract

Susanne Kaesler, Désirée Argiriu, Shyami M. Kandage, Karla Schönfeldt, Shalva Lekiashvili, Ceren N. Dengiz, Neslim Ercan1, Caterina Iuliano, Martina Herrmann, Maria Reichenbach; Dominik Cichowski, Magda Babina, Miriam Hils, Martin Köberle, and Tilo Biedermann

Supplementary Method S1

β-arrestin path-hunter assay

The PathHunter® eXpress MRGPRX2 CHO-K1 β-Arrestin GPCR Assay was performed by Eurofins DiscoverX (Fremont, CA, USA). The assay is based on the complementation of two enzyme fragments, of which one is fused to MRGPRX2 and the other to β-arrestin. Ligand-binding induced MRGPRX2 activation and β-arrestin recruitment results in a complementation of a functional enzyme whose substrate conversion produces light emission. This assay was used to determine the effect of oat extract on MRGPRX2 activation and β-arrestin recruitment. Briefly, CHO-K1 cells were incubated for 30 minutes at 37°C with increasing doses of oat extract before S.P. was added at the effective concentration (EC)80 (1.89µM). Chemiluminescent signal detection was performed with a PerkinElmer Envision instrument.

Supplementary Figure S1

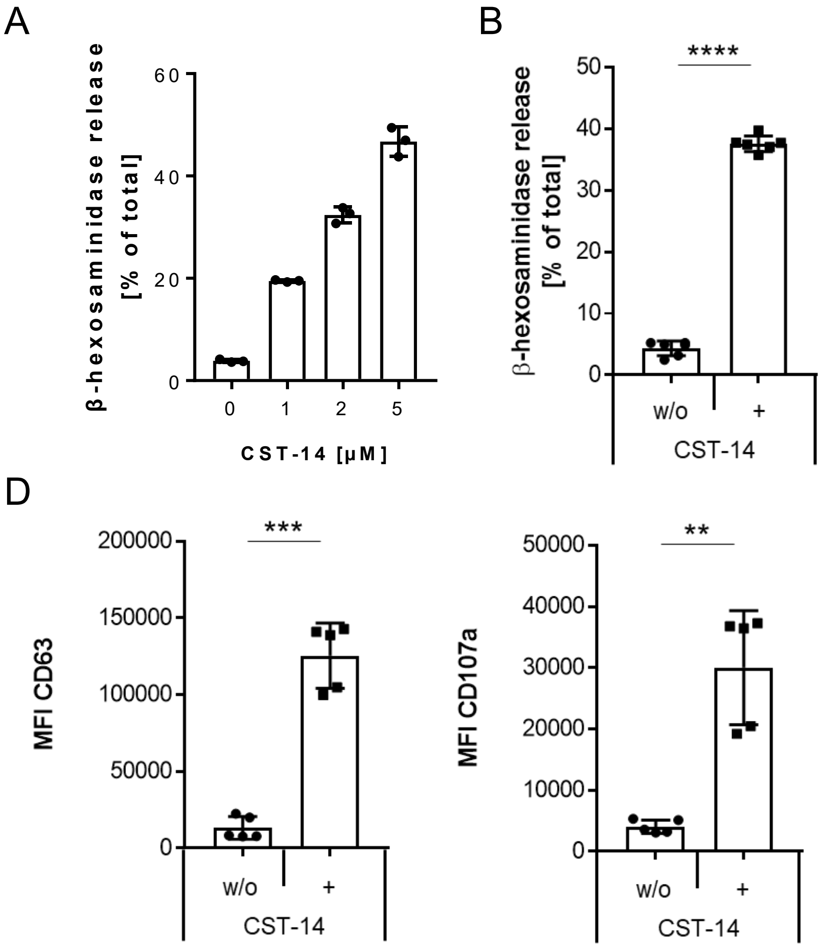

Supplementary Figure s1: MRGPRX2 activation by the neuropeptide cortistatin 14 in human mast cell line LAD2

(**A**) Dose-dependent β-hexosaminidase release upon stimulation of LAD2 cells with CST-14 (one of two experiments, each with triplicates). (**B -D**) Degranulation of LAD2 cells after stimulation with 5µM CST-14 as shown by β-hexosaminidase (**B**), tryptase (**C**), and MFIs of CD63 and CD107a (**D**). Summary of 6 (B) or 5 (C, D) experiments with single values or duplicates are shown.

Supplementary Figure S2

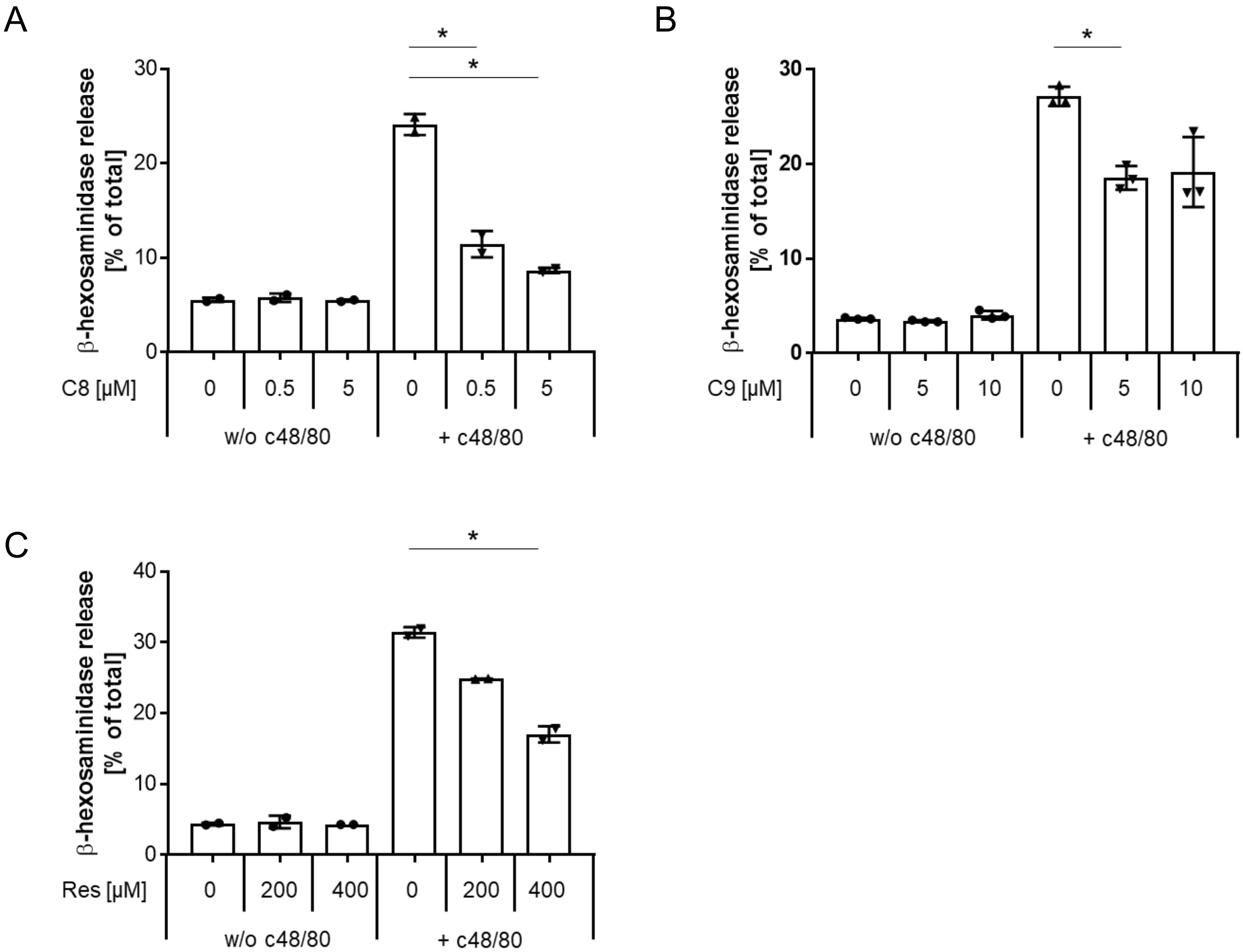

Supplementary Figure S2: Titration of MRGPRX2 antagonists in LAD2 cells
LAD2 cells were exposed to different concentrations of known MGRPRX2 inhibitors C8 (**A**), C9 (**B**), and resveratrol (**C**) before they were stimulated with c48/80. Degranulation is assessed by β-hexosaminidase release. One representative experiment of two is shown.

Supplementary Figure S3

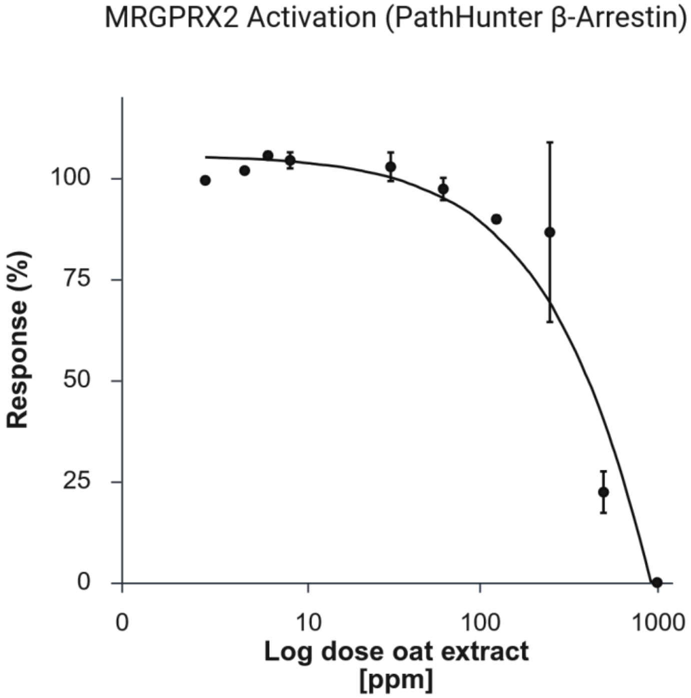

Supplementary Figure S3: Oat extract inhibits MRGPRX2–mediated ß-arrestin recruitment in CHO-K2 cells

Cho-K2 cells overexpressing a modified MRGPRX2 were stimulated with EC80 S.P. (1.89μM) and increasing amounts of oat extract. MRGPRX2 activation induced ß-arresting recruitment was assessed by detection of luminescence generated by enzyme fragment complementation using the PathHunter ß-arresting assay.

Supplementary Figure S4

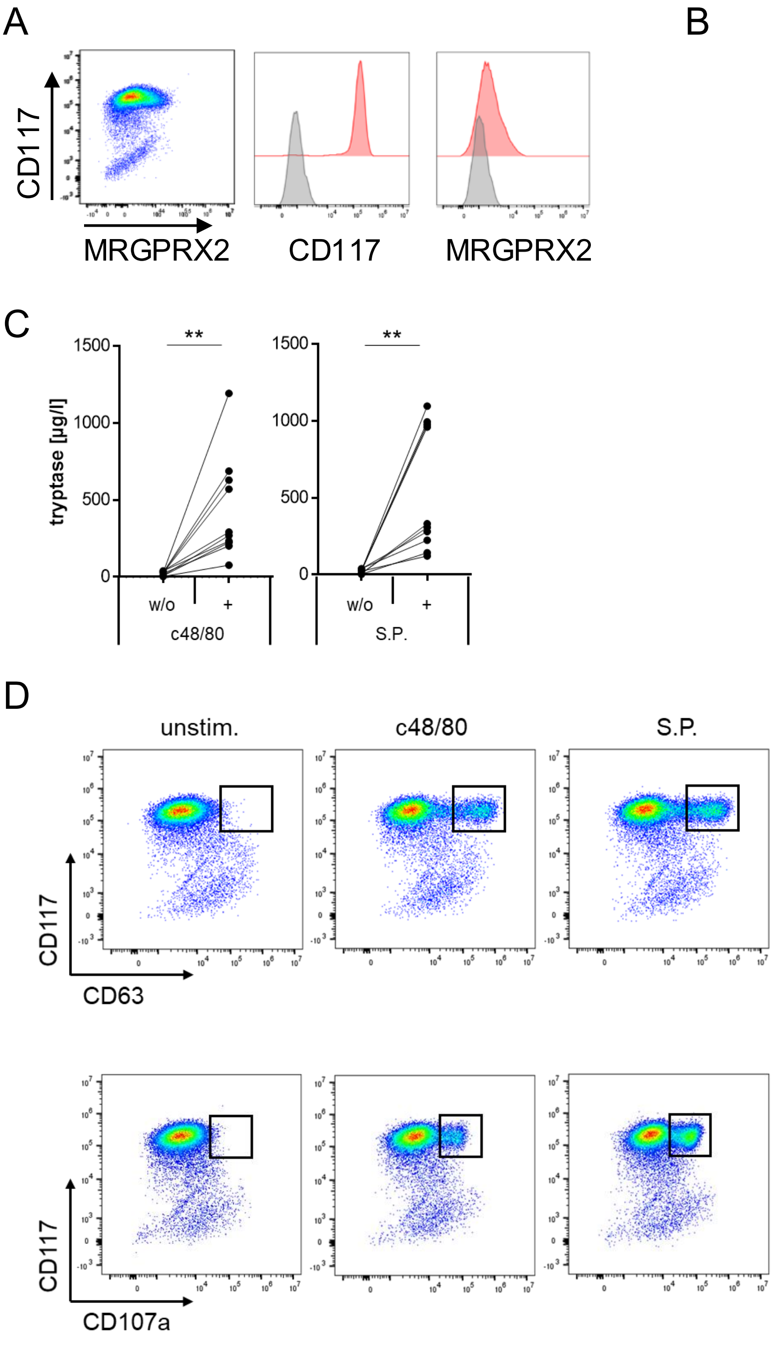

Supplementary Figure S4: Activation of MRGPRX2 in primary human blood-derived mast cells (BDMC)

(**A**) Representative dot plot and histograms showing the expression of MC receptors CD117 and MRGPRX2 in BDMCs. (**B-E**) BDMCs were stimulated with 5µg/ml c48/80 or 10µM S.P. and degranulation was assessed compared to unstimulated cells by β-hexosaminidase (**B**), tryptase (**C**), and CD63 and CD107a expression shown as dot plots (**D**, representative example), or MFI (**E**). Summary of experiments performed with BDMC from 24 or 19 (B), 10 (C) or 11 (E) donors.

Supplementary Figure S5

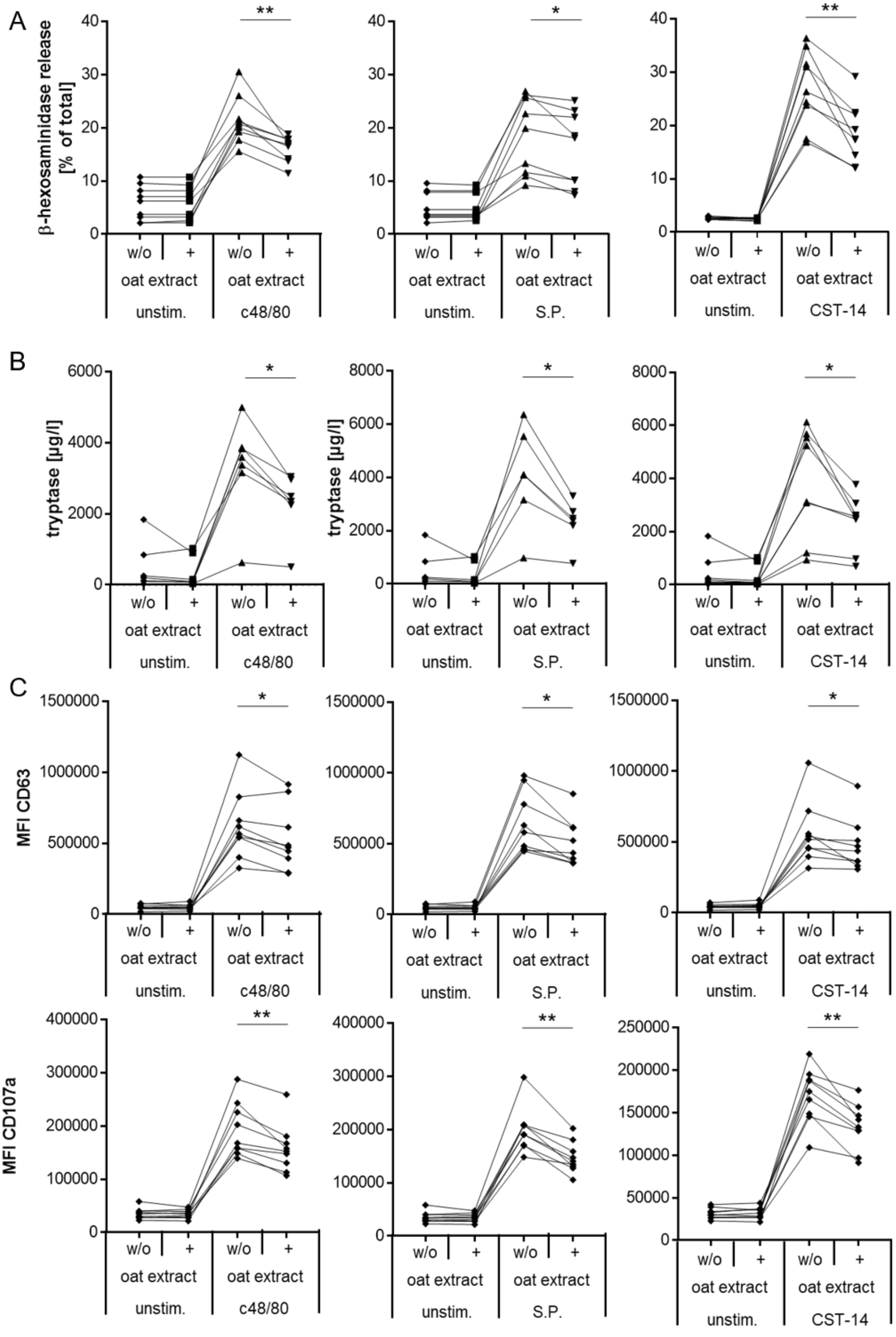

Supplementary Figure S5: Inhibition of MRGPRX2 activation in primary human BDMC by oat extract

Primary BDMCs were pre-exposed to oat extract followed by stimulation with different MRGPRX2 ligands. (**A**) β-hexosaminidase release, (**B)** tryptase release; (**C**), changes in surface exposure of CD63 and CD107a. Summary of experiments from 9 (A), 6-8 (B) or 9 (C) donors are shown
